# Attention and positive affect: Temporal switching or spatial broadening?

**DOI:** 10.3758/s13414-015-0845-1

**Published:** 2015-03-14

**Authors:** R. Hans Phaf

**Affiliations:** 1 Amsterdam Brain and Cognition Center, University of Amsterdam, Amsterdam, The Netherlands; 2 Brain and Cognition Group, Department of Psychology, University of Amsterdam, Weesperplein 4, 1018 XA Amsterdam, The Netherlands

**Keywords:** Attention, Selective, Temporal Processing, Attention, Theoretical and Computational Models

## Abstract

Evolutionary reasoning and computation suggest that positive affect is associated with higher attentional flexibility than negative affect, even when affectively neutral material is processed. The affective modulation of interference in the Eriksen flanker task seems, however, more readily explained by a spatial broadening of attention due to positive affect. It is argued here that these results should also be interpreted in terms of an increased switching over time between flankers and target (i.e., flexibility). The two hypotheses were contrasted with positive and negative mood inductions in a masked-flanker task. The interval (Stimulus Onset Asynchrony; SOA) with which the masked flankers preceded the target letter was parametrically varied. In contrast to what is found with simultaneous non-masked flanker presentation, masking produced larger interference with negative than with positive moods. In addition, a crossover interaction between mood and SOA emerged. These results seem incompatible with a spatial broadening account and support an affective modulation account in terms of flexibility.

## Introduction

Evolution has equipped the human organism with basic mechanisms for dealing with opportunities and priorities (see Phaf & Rotteveel, 2012). These mechanisms are often labeled emotion and attention, which seem closely linked because the former determines what receives priority, and the latter how it is given (cf. Compton, 2003). In our evolutionary simulations it serendipitously emerged that positive affect was associated with high flexibility and facilitated switching over time to potentially negative events, and that negative affect corresponded with low distractibility (Heerebout & Phaf, 2010a, b). This hypothesis agrees with the classic broaden-and-build theory due to positive affect of Fredrickson (2004), which however compounds modulatory effects on temporal (i.e., flexibility) and spatial (i.e., the focus size) attention. Although no evolutionary motivation for it has been posited, the broadening hypothesis holds that positive affect extends spatial attention to large-scale stimulus features. Both flexibility (e.g., Baumann & Kuhl, 2005; Heerebout, Todorović, Smedinga, & Phaf, 2013; Tan, Jones, & Watson, 2009; but see Huntsinger, Clore, & Bar-Anan, 2010) and broadening hypotheses (Gasper & Clore, 2002; Rowe, Hirsh, & Anderson, 2007) have received ample empirical support. This study aims to show that one of the findings supporting the spatial hypothesis is more consistent with the temporal hypothesis.

Evolutionary development of neural networks can be simulated computationally by subjecting artificial neural networks to random variation (i.e., mutations and crossovers) and selecting those networks for reproduction that are most adapted to their environment. Heerebout and Phaf (2010a, b) simulated agents controlled by such evolving networks that wandered in an artificial world containing food patches and predators. From a random start, selection of the fittest (i.e., living the longest and gathering the most food) led to the autonomous development of organized behavior (i.e., food approach and predator avoidance) and specific network architectures (e.g., mutually inhibitory, competitive networks). Interestingly, oscillations of node activations in the networks also emerged which corresponded to a near-doubling of fitness, indicating that they had a clear adaptive function (Heerebout & Phaf, 2010a). The low-activation, low-inhibition troughs in the oscillations provided opportunities for switching winners in the competition between stimulus representations. The hypothesis that it is more adaptive to switch attention when the agent is gathering food than when fleeing predators was also supported by the results. The oscillations had a higher frequency in the former case than in the latter (Heerebout & Phaf, 2010b). From this finding we postulated that positive affect (i.e., coding with high frequencies for fitness-enhancing conditions) correspond to a higher flexibility than negative affect (i.e., coding with low frequencies for fitness-reducing conditions) in biological networks too.

A suitable paradigm for investigating the affective modulation of attention is the classic Eriksen flanker task (Eriksen & Eriksen, 1974). Here adjacent, congruent (e.g., N N N N N) or incongruent (e.g., H H N H H) letters influence the time to classify the central letter. In support of spatial broadening, Rowe and collaborators (2007; but see Bruyneel, van Steenbergen, Hommel, Band, De Raedt, & Koster, 2013) found that positive music enhanced interference by incompatible flankers relative to neutral and sad inductions for near, medium, and far flanker-target distances. Furthermore, the interference appeared to decrease as a function of distance with the latter inductions. This fits well within an attentional selection by neural competition view (e.g., Duncan, 1996; Phaf, van der Heijden, & Hudson, 1990) by assuming that the spatial range of competition (i.e., the range of inhibition between nodes representing different positions) is extended by positive affect. In an accompanying remote-associate task in the Rowe et al. (2007) study, access to remote associates was also facilitated by positive affect. In addition, flanker interference correlated across participants with remote-associate performance after the positive mood induction.

It is argued here that positive affect enhances flanker interference by increasing attentional switching between target and flankers. Both spatial broadening and temporal flexibility can be envisaged within a competitive framework. The former entails a broadening of the inhibition range, whereas the latter involves switching winning node activations (for a more detailed description, see Heerebout & Phaf, 2010a, b). In addition, the evolutionary simulations suggest that it does not involve high-level control processes (cf. Dreisbach, 2006) but rather concerns basic modulatory effects on attention. In a similar fashion to the simulations, in the Eriksen flanker task positive affect should automatically increase switching between flankers and target, which are both presented until response, and thus cause greater interference. Because semantic associates do not have an obvious spatial arrangement, a flexibility account (i.e., switching between associates) also seems more appropriate for the remote-associate task than the broadening hypothesis.

The aim of this study was to experimentally distinguish flexibility and broadening hypotheses by limiting through flanker masking the number of switches made between flankers and target. Schwarz and Mecklinger (1995) obtained interference by masked flankers presented for as little as 28 ms, even when there was no evidence of conscious flanker perception. With the shorter duration of 14 ms, however, the interference disappeared. For longer durations it increased as a function of duration, possibly due to the larger opportunity for switching. In a neutral mood the 28-ms presentation duration apparently provided just sufficient time to switch between the perceptual representations of flanker and simultaneously presented target. This does not necessarily mean that the switch is made within 28 ms, because the mask does not immediately turn off perceptual activations. If positive affect does indeed increase switching speed, this shortens the time spent at the flanker. A faster disengagement from an incongruent flanker with positive than with negative affect would then predict a reversal with respect to the Rowe et al. (2007) findings, resulting in more interference in negative than in positive conditions. This contrasts sharply with the prediction derived from the broadening account, where the spatial range of interference should not be influenced by the masking.

Giving the (unmasked) flankers a head-start enhanced interference in the absence of mood inductions (Eriksen & Schultz, 1979; Flowers, 1980). The larger opportunity for scanning identical flankers presumably leads to a larger build-up of competing flanker activation. This may even be the case with masking, because the switch is made here between identical flankers instead of between flanker and target. It should be noted that this build-up may extend over longer intervals than nominal flanker duration because of the longer persistence of the perceptual representations that are subject to switching. If flankers are scanned more frequently with positive than with negative affect, this may again result in a larger build-up of competing flanker activation and thus a larger interference with positive affect when flanker-target interval (i.e., Stimulus Onset Asynchrony, SOA) lengthens. Indeed, Schmidt, Haberkamp, and Schmidt (2011) recommended such parametric variations in what they called response priming research, because they “…can reveal unexpected nonlinearities (e.g., a change of sign in the dependent variable).” (p. 123)

The experiment consisted of an Eriksen masked-flanker task and a masked flanker-identification block, which could serve as a check for conscious flanker processing (cf. Schwarz & Mecklinger, 1995). The primary aim of masking here, however, was to limit the number of switches and not to demonstrate a dissociation between conscious and non-conscious processing, as in Schwarz and Mecklinger (for a critique on the dissociation procedure, see Merikle, 1992). The Eriksen and identification blocks were performed after a positive induction and after a negative induction, in counterbalanced order. To induce these moods, participants were asked to write about positive and negative personal events, while listening to pre-selected positive and negative music. This combined induction procedure seems particularly effective in enhancing positive affect (Zhang, Yu, & Barrett, 2014). To check for mood level, subjective reports on mood state were requested at the start of the experiment and after positive and negative inductions.

## Method

### Participants

Forty psychology students (mean age 21.0 ± 1.42 years; 25 female) from the University of Amsterdam with normal or corrected-to-normal vision participated for either course credit or a financial compensation, after providing signed informed consent. Participants who made more than 10 % errors on the easy letter-detection task were removed from the analyses.

### Design

The flanker task had a 2 × 2 × 5 within-participants factorial design. Mood induction (positive and negative) served as the first independent variable. The order of inductions was counterbalanced across participants. The second independent variable concerned the flanker-target compatibility (e.g., incongruent: HHNHH; congruent: HHHHH). Finally, SOA between flanker and target had five levels (i.e., 0, 30, 60, 90, and 120 ms). Trial order was determined randomly by the computer with number of trials per condition remaining equal. The SOA manipulation was irrelevant in the flanker identification task, which consequently had a 2 × 2 design.

Reaction time (RT) to correct responses served as the dependent variable in the flanker task. Outliers’ RTs outside the 2.5 SD range from the condition mean were removed. Error proportions were also recorded, but not analyzed separately due to their low level (see Table 1). To adjust for different strategies in the two moods, RTs and errors were combined in Inverse Efficiency Scores (IES = RT/Accuracy; Townsend & Ashby, 1978). Smilek, Enns, Eastwood, and Merikle (2006) argued that IES can also serve as a correction for speed-accuracy trade-offs. Because average RT is divided by the proportion correct per condition and per participant, RTs of slow, accurate, participants generally remain the same, and those of fast, less accurate, participants are somewhat elevated. At the low error level and high correlation between RT and errors across conditions (in this study *r* = 0.762, *p* < 0.0001) for which this score is deemed appropriate (Bruyer & Brysbaert, 2011), IES can be considered a kind of RT adjusted for strategy and speed-accuracy trade-off. A flanker-compatibility index (FCI) was calculated per condition by subtracting congruent IES from incongruent IES (FCI = IES_incongruent_ – IES_congruent_). The dependent variable with flanker-identification was the proportion of correct identifications of the flankers.

**Table 1:** Reaction times (RTs) in ms, percentage correct (Pc), and Inverse Efficiency Scores (IES) in ms in the flanker task. SDs are given in parentheses

Mood	Compatibility	SOA														
		0			30			60			90			120		
		RT	Pc	IES	RT	Pc	IES	RT	Pc	IES	RT	Pc	IES	RT	Pc	IES
Positive	Congruent	402 (49)	97.9 (3.5)	412 (53)	408 (52)	98.1 (3.1)	418 (60)	391 (57)	98.8 (1.8)	396 (62)	382 (48)	98.4 (2.5)	389 (50)	377 (48)	98.1 (2.5)	385 (47)
	Incongruent	424 (47)	95.8 (3.5)	444 (50)	444 (46)	95.3 (4.3)	466 (48)	435 (52)	93.2 (5.7)	468 (56)	429 (50)	94.1 (4.7)	457 (52)	417 (47)	95.0 (5.9)	441 (47)
Negative	Congruent	408 (46)	98.1 (2.2)	416 (48)	417 (50)	98.5 (2.1)	423 (52)	402 (47)	98.5 (2.2)	409 (50)	395 (45)	99.0 (1.5)	400 (45)	393 (49)	99.0 (1.6)	398 (51)
	Incongruent	439 (58)	95.8 (5.1)	463 (62)	456 (45)	95.6 (4.2)	478 (51)	444 (46)	96.4 (3.1)	461 (47)	438 (47)	96.1 (4.5)	457 (51)	431 (45)	95.0 (3.5)	454 (46)

*SOA* Stimulus Onset Asynchrony

### Materials and apparatus

The stimuli were presented against a light-gray background in a dimly lit room on a 23-in Asus VG246HE monitor with a 1920 × 1080 resolution and 100 Hz refresh rate. Distance from the screen was approximately 60 cm. Music was played on Sennheiser HD 201 headphones from an iPod classic 120 GB. Reaction times were registered from a two-button response box. Corresponding to the QWERTY keyboard, the left button represented the letter H and the right button the N. Participants were instructed to hold their dominant hand on the response box and to use their index and middle fingers for button pressing.

Both flanker and target stimuli consisted of the black letters H or N (Calibri font, 100 point size). Flankers were positioned directly adjacent to the central target. Flankers were masked by random dot patterns (154 × 84 pixel). Every 4 × 4 pixel block in the mask had a 50 % chance of being black or white. Moods were induced by instructing the participant to write about a positive or negative personal event, while listening to positive or negative music fragments they had selected beforehand (see Appendix 1). The music fragments were looped through the induction and experimental phases. At the start and after each induction, participants were asked to indicate their mood on a 1 (extremely negative; left) to 9 (extremely positive; right) mood-scale.

### Procedure

**Fig. 1:**
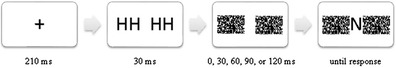
Timeline of an incongruent trial in the flanker task

Participants were informed that the experiment investigated the influence of mood on attentional processing, and that this would involve a mood induction procedure and a few simple computer tasks. They first indicated their baseline mood level on the mood scale, and performed ten practice trials. A flanker trial (see Fig. 1) started with a fixation cross presented for 210 ms. Subsequently, flankers on both sides of the fixation help appeared and were masked after 30 ms. Flankers were followed by the central target after an SOA of 0, 30, 60, 90, or 120 ms. Target and masks remained on the screen until response. The intertrial interval was 1500 ms with a random jitter of ± 500 ms. The flanker identification task was similar to the masked flanker task but did not contain the central target letter. Instead of responding to the central letter, participants now indicated whether the briefly presented flankers were the letters ‘N’ or ‘H’. Participants were instructed to respond as quickly and accurately as possible, because RTs and errors were registered.

For the induction participants were instructed to write about either a positive or a negative personal event for a period of 4 min. They were first asked to select a corresponding music fragment, which would be played during induction and flanker and identification tasks. They were told that their writing would not be read by the experimenter and that they should take it home. After completing the mood induction, participants were again asked to indicate their mood. Subsequently, participants performed the flanker task (400 trials per induction block) and the identification task (80 trials per induction block). Ten practice trials again preceded the identification task. Between the two parts of the experiment with the opposite mood inductions, participants took a short break. In the exit interview, participants were asked for impressions and possible strategies used during the experiment. At the end the participant’s mood was checked, and if it seemed too negative, attempts were made to return it to the original mood state.

## Results

Four participants were excluded because three made more than 10 % letter-detection errors and one reported not having followed instructions. Differential moods were successfully induced according to the participants’ reports. All mood reports were biased to the positive side. The baseline mood (*M* = 6.96 ± 0.88; one participant report was lost) differed from the neutral midpoint 5 (*t*(34) = 13.194, *p* < 0.0001). Even the negative induction showed more positive reports (*M* = 5.51 ± 1.03) than midpoint (*t*(35) = 2.056, p < 0.05), as did the positive induction (*M* = 7.35 ± 1.50; two participant reports were lost; *t*(33) = 13.367, *p* < 0.0001). All mood reports differed significantly from each other (*p*s < 0.05). The order of inductions did not reveal a reliable effect on any of the dependent variables in this study.

### Masked flanker task

After removal of incorrect responses and outliers, 87.9 % of trials remained available for analysis. The results of the Eriksen Flanker task are presented in Table 1.

Despite flanker masking, letter detection was consistently faster with congruent (IES = 405 ± 53 ms) than with incongruent (IES = 459 ± 52 ms) flankers. This was confirmed by a main effect in the 2 × 2 × 5 ANOVA (*F*(1, 35) = 266.62, *p* < 0.0001, η_p_^2^ = 0.884). In absolute terms, positive moods produced faster responses (IES = 428 ± 60 ms) than negative moods (IES = 436 ± 57 ms), but this was not significant (*F*(1, 35) = 2.354, *p* = 0.134, η_p_^2^ = 0.06). The SOA effect (*F*(4, 140) = 24.188, *p* < 0.0001, η_p_^2^ = 0.408) was qualified by an interaction with compatibility (*F*(4, 140) = 7.019, *p* < 0.0001, η_p_^2^ = 0.167) and by a triple interaction with compatibility and mood (*F*(4, 140) = 4.208, *p* < 0. 005, η_p_^2^ = 0.107). The latter interaction was only driven by target-flanker competition as was revealed by a significant SOA × Induction interaction only in incongruent conditions (*F*(4, 140) = 3.567, *p* < 0.001, η_p_^2^ = 0.093).

**Fig. 2:**
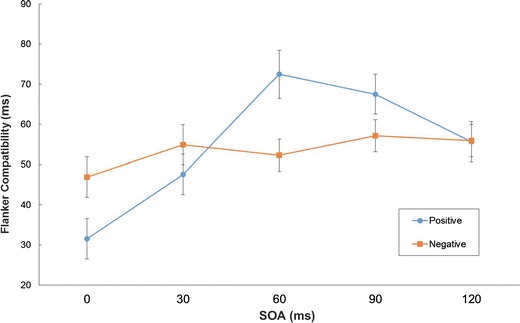
Average flanker-compatibility index in ms as a function of Stimulus Onset Asynchrony (SOA) and mood. Error bars denote 1 standard error (SE), and have been adjusted for within-subjects designs

To further elucidate the interactions, the FCI was calculated (see Fig. 2). Interference increased as a function of SOA (SOA = 0 ms: FCI = 39 ± 32 ms; SOA =30 ms: FCI = 51 ± 37 ms; SOA = 60 ms: FCI = 62 ± 41 ms; SOA = 90 ms: FCI = 62 ± 35; SOA = 120 ms: FCI = 56 ± 30 ms). More importantly, the crossover interaction meant that at short SOAs (SOA = 0 ms: *t*(35) = 2.290, *p* < 0.05, Cohen's *d* = 0.48) interference was larger with negative than with positive moods, but was smaller for longer SOAs (SOA = 60 ms: *t*(35) = −2.780, *p* < 0.01, *d* = 0.51; SOA = 90 ms: *t*(35) = −1.701, *p* = 0.098, *d* = 0.29). For SOA = 30 ms and SOA = 120 ms the mood conditions did not differ reliably. Only positive inductions modulated flanker interference across SOAs (*F*(4, 140) = 10.062, *p* < 0. 0001, η_p_^2^ = 0.223).

### Identification task

Participants showed substantial recognition of flankers, and slightly, but not significantly, more so in positive (*M* = 0.564 ± 0.118) than in negative (*M* = 0.550 ± 0.134) conditions. With both positive (*t*(35) = 28.748, *p* < 0.001) and negative (*t*(35) = 24.560, *p* < 0.001) inductions, performance was significantly above chance level (0.5).

## Discussion

The affective modulation of flanker interference not only depended on but even reversed as a function of flanker-target interval. This implies that there must be at least a temporal component to these mood effects. Affective modulation of only spatial attention could correspond to a strengthening or weakening of interference as a function of interval (e.g., due to a decay of flanker activations), but not to a complete reversal. At SOA = 0 ms, moreover, more interference was found with negative than with positive moods. Rowe et al. (2007), who presented non-masked flankers until response, obtained the opposite pattern of affective modulation. It should be noted that in the present study mood reports were above the neutral midpoint even after the negative induction, which may be due to positive appreciation of even the negative music. The comparisons here were thus made between positive and less positive conditions and tell us more about the impact of positive moods rather than negative moods. In addition, the much larger variation in flanker interference across SOAs after positive than after negative inductions may also indicate that positive affect primarily drives these modulatory effects.

The crucial factor in the contrast at SOA = 0 ms between these results and the findings of Rowe et al. (2007; but see Bruyneel et al., 2013) is the masked flanker presentation. The results of Schwarz and Mecklinger (1995) suggest that in a neutral mood with simultaneous presentation and masked flankers (i.e., after 30 ms) only one switch can be made. Disengagement from the flanker should then be facilitated, less time spent at the flanker, less competing flanker activation accumulated, and interference reduced by positive affect. The opposite increased difficulty of disengaging due to negative affect suggests a flexibility-rigidity continuum in affective modulation. This has been well documented not only in (sub-)clinically anxious persons with threatening material (e.g., Fox, Russo, Bowles, & Dutton, 2001; Phaf & Kan, 2007), but also in non-clinical participants with negative material (e.g., Belopolsky, Devue, & Theeuwes, 2011; Most, Chun, Widders, & Zald, 2005; Peltola, Leppänen, Vogel-Farley, Hietanen, & Nelson, 2009), and even with neutral material (e.g., Baumann & Kuhl, 2005; Heerebout et al., 2013). In addition, the flexibility account is supported here by the crossover interaction of mood and SOA. At non-zero SOAs when the target is not presented yet, the positive affect facilitates scanning between identical flankers, and thus promotes the buildup of activation competing with the future target.

Even though flankers were masked, an appreciable interference effect was found in all conditions. The flankers in the Schwarz and Mecklinger (1995) study were not reliably identified when presented for 28 ms. This result was not replicated here with masked presentation for 30 ms. Masking was included, however, not for obtaining dissociations between conscious and non-conscious processing (Merikle, 1992), but to limit the number of attentional switches between the perceptual representations of flankers and target. Similar to Eriksen and Schultz (1979; see also Flowers, 1980), here too interference increased in positive conditions, particularly from SOA = 0 ms to SOA = 60 ms, but unexpectedly decreased again with SOAs longer than 60 ms. Both spatial broadening and temporal switching could be invoked to account for these increases and decreases at non-zero SOAs (e.g., by adding accumulation and decay mechanisms), but only the latter allows for a change of sign in the affective modulation as a function of SOA.

The Eriksen Flanker task constitutes a further paradigm after the local-global task where the modulatory effects on processing affectively neutral stimuli can be reduced from a broadening (Gasper & Clore, 2002) to a flexibility account (Baumann & Kuhl, 2005; Huntsinger et al., 2010; Tan et al., 2009). In a large number of other tasks, higher flexibility due to positive affect has been observed (e.g., semantic remote-associates task: Bolte, Goschke, & Kuhl, 2003; Rowe et al., 2007; creative problem solving: Isen, Daubtman, & Nowicki, 1987; control processes: Dreisbach & Goschke, 2004; Dreisbach, 2006; De Vries, Holland, Corneille, Rondeel, &Witteman, 2012). Higher retrieval flexibility also lured participants into more false memories in the Deese-Roediger-McDermott paradigm with positive than with negative affect (Storbeck & Clore, 2005). These results cannot preclude that the broadening account is still valid for other paradigms, but they add to our evolutionary simulations (Heerebout & Phaf, 2010ab) and strengthen the notion (see also, Heerebout et al., 2013) that the modulation of flexibility is the most basic, and evolutionary early, effect of positive affect.

## Appendix 1

Table 2

**Table 2:** List of positive and negative music fragments the participants could choose from

Positive music	Negative music
Daft Punk – Get Lucky	Johnny Cash – Hurt
Pharrell Williams – Happy	Sia – Breathe Me
Netsky – Puppy	Samuel Barber – Adagio for Strings
Bob Marley – Don’t Worry be Happy	Portishead – Roads
Prodigy – Stand Up	Yann Tiersen – Comptine d’un autre été

## References

[CR1] Baumann N, Kuhl J (2005). Positive affect and flexibility: Overcoming the precedence of global over local processing of visual information. Motivation and Emotion.

[CR2] Belopolsky AV, Devue C, Theeuwes J (2011). Angry faces hold the eyes. Visual Cognition.

[CR3] Bolte A, Goschke T, Kuhl J (2003). Emotion and intuition: Effects of positive and negative mood on implicit judgments of semantic coherence. Psychological Science.

[CR4] Bruyer R, Brysbaert M (2011). Combining speed and accuracy in cognitive psychology: Is the Inverse Efficiency Score (IES) a better dependent variable than the mean Reaction Time (RT) and the Percentage of Errors (PE)?. Psychologica Belgica.

[CR5] Bruyneel L, van Steenbergen H, Hommel B, Band GP, De Raedt R, Koster EH (2013). Happy but still focused: Failures to find evidence for a mood-induced widening of visual attention. Psychological Research.

[CR6] Compton RJ (2003). The interface between emotion and attention: A review of evidence from psychology and neuroscience. Behavioral and Cognitive Neuroscience Reviews.

[CR7] De Vries M, Holland RW, Corneille O, Rondeel E, Witteman CLM (2012). Mood effects on dominated choices: Positive mood induces departures from logical rules. Journal of Behavioral Decision Making.

[CR8] Dreisbach G (2006). How positive affect modulates cognitive control: The costs and benefits of reduced maintenance capability. Brain & Cognition.

[CR9] Dreisbach G, Goschke T (2004). How positive affect modulates cognitive control: Reduced perseveration at the cost of increased distractibility. Journal of Experimental Psychology: Learning, Memory, and Cognition.

[CR10] Duncan, J. (1996). Cooperating brain systems in selective perception and action. In T. Inui & J. L. McClelland (Eds.), *Attention and performance XVI: Information integration in perception and communication* (pp. 549–578). Cambridge, MA: MIT Press.

[CR11] Eriksen BA, Eriksen CW (1974). Effects of noise letters upon the identification of a target letter in a non-search task. Perception & Psychophysics.

[CR12] Eriksen CW, Schultz DW (1979). Information processing in visual search: A continuous flow conception and experimental results. Perception & Psychophysics.

[CR13] Flowers JH (1980). Response priming effects in a digit naming task as a function of target-noise separation. Bulletin of the Psychonomic Society.

[CR14] Fox E, Russo R, Bowles R, Dutton K (2001). Do threatening stimuli draw or hold visual attention in subclinical anxiety?. Journal of Experimental Psychology: General.

[CR15] Fredrickson BL (2004). The broaden-and-build theory of positive emotions. Philosophical Transactions of the Royal Society B: Biological Sciences.

[CR16] Gasper K, Clore GL (2002). Attending to the big picture: Mood and global versus local processing of visual information. Psychological Science.

[CR17] Heerebout BT, Phaf RH (2010). Emergent oscillations in evolutionary simulations. Journal of Cognitive Neuroscience.

[CR18] Heerebout BT, Phaf RH (2010). Good vibrations switch attention: An affective function for network oscillations in evolutionary simulations. Cognitive, Affective, & Behavioral Neuroscience.

[CR19] Heerebout BT, Todorović A, Smedinga HE, Phaf RH (2013). Affective modulation of attentional switching. The American Journal of Psychology.

[CR20] Huntsinger JR, Clore GL, Bar-Anan Y (2010). Mood and global–local focus: Priming a local focus reverses the link between mood and global–local processing. Emotion.

[CR21] Isen AM, Daubman KA, Nowicki GP (1987). Positive affect facilitates creative problem solving. Journal of Personality and Social Psychology.

[CR22] Merikle PM (1992). Perception without awareness. American Psychologist.

[CR23] Most SB, Chun MM, Widders DM, Zald DH (2005). Attentional rubbernecking: Cognitive control and personality in emotion-induced blindness. Psychonomic Bulletin & Review.

[CR24] Peltola MJ, Leppänen JM, Vogel-Farley VK, Hietanen JK, Nelson CA (2009). Fearful faces but not fearful eyes alone delay attention disengagement in 7-month-old infants. Emotion.

[CR25] Phaf RH, Kan KJ (2007). The automaticity of emotional Stroop: A meta-analysis. Journal of Behavior Therapy and Experimental Psychiatry.

[CR26] Phaf RH, Rotteveel M (2012). Affective monitoring: A generic mechanism for affect elicitation. Frontiers in Psychology.

[CR27] Phaf RH, Van der Heijden AHC, Hudson PTW (1990). SLAM: A connectionist model for attention in visual selection tasks. Cognitive Psychology.

[CR28] Rowe G, Hirsh JB, Anderson AK (2007). Positive affect increases the breadth of attentional selection. Proceedings of the National Academy of Sciences.

[CR29] Schmidt F, Haberkamp A, Schmidt T (2011). Do’s and don’ts in response priming research. Advances in Cognitive Psychology.

[CR30] Schwarz W, Mecklinger A (1995). Relationship between flanker identifiability and compatibility effect. Perception & psychophysics.

[CR31] Smilek D, Enns JT, Eastwood JD, Merikle PM (2006). Relax! Cognitive strategy influences visual search. Visual Cognition.

[CR32] Storbeck J, Clore GL (2005). With sadness comes accuracy; With happiness, false memory. Psychological Science.

[CR33] Tan HK, Jones GV, Watson DG (2009). Encouraging the perceptual underdog: Positive affective priming of nonpreferred local-global processes. Emotion.

[CR34] Townsend JT, Ashby FG, Castellan NJ, Restle F (1978). Methods of modeling capacity in simple processing systems. Cognitive theory.

[CR35] Zhang X, Yu HW, Barrett LF (2014). How does this make you feel? A comparison of four affect induction procedures. Frontiers in Psychology.

